# Design of High Volume CFBC Fly Ash Based Calcium Sulphoaluminate Type Binder in Mixtures with Ordinary Portland Cement

**DOI:** 10.3390/ma14195798

**Published:** 2021-10-03

**Authors:** Peeter Paaver, Oliver Järvik, Kalle Kirsimäe

**Affiliations:** 1 Department of Geology, Institute of Ecology and Earth Sciences, University of Tartu, Ravila 14A, 50411 Tartu, Estonia; kalle.kirsimae@ut.ee; 2 Department of Energy Technology, Tallinn University of Technology, Ehitajate tee5, 19086 Tallinn, Estonia; oliver.jarvik@taltech.ee

**Keywords:** fly ash grinding, compressive strength, ettringite, calcium sulphoaluminate cement, waste utilisation

## Abstract

Growing concerns on global industrial greenhouse gas emissions have boosted research for developing alternative, less CO_2_ intensive binders for partial to complete replacement of ordinary Portland cement (OPC) clinker. Unlike slag and pozzolanic siliceous low-Ca class F fly ashes, the Ca- and S-rich class C ashes, particularly these formed in circulating fluidised bed combustion (CFBC) boilers, are typically not considered as viable cementitious materials for blending with or substituting the OPC. We studied the physical, chemical-mineralogical characteristics of the mechanically activated Ca-rich CFBC fly ash pastes and mortars with high volume OPC substitution rates to find potential alternatives for OPC in building materials and composites. Our findings indicate that compressive strength of pastes and mortars made with partial to complete replacement of the mechanically activated CFBC ash to OPC is comparable to OPC concrete, showing compared to OPC pastes reduction in compressive strength only by <10% at 50% and <20% at 75% replacement rates. Our results show that mechanically activated Ca-rich CFBC fly ash can be successfully used as an alternative CSA-cement type binder.

## 1. Introduction

Production of ordinary Portland cement (OPC) emits a large fraction, about 8%, of the anthropogenic greenhouse gases [1]. Therefore, finding alternative raw materials, controlling the carbon dioxide emissions from the binder’s production, developing substituting and/or supplementary cementitious materials, and designing alternative low-carbon binders have become the priorities of the cement industry [2]. Calcium sulphoaluminate (CSA) and Ca-sulphoaluminate-belite (CSA-C2S) cements are increasingly coming into focus as sustainable, less CO_2_ intensive alternatives to OPC [3,4,5,6,7,8]. CSA cements exhibit mechanical-chemical properties similar or even exceeding that of the OPC—e.g., in terms of rapid strength development, low shrinkage and chemical durability—that has allowed their use in different construction applications replacing or along with OPC [5,9,10,11], but also in geotechnics for weak soil stabilisation [12,13].

CSA type cements have been widely used in China, where CSA cement was developed more than 40 years ago [3]. CSA cements are composed mainly of ye’elimite [C4A3-Ca_4_(AlO_2_)_6_SO_3_)], belite (C2S—dicalcium silicate), and gypsum (CaSO_4_ 2H_2_O), and the main hydration products providing the cementation are ettringite (C6A2H26-Ca_6_Al_2_(OH)_12_(SO_4_)_3_·26H_2_O) and nanocrystalline Al(OH)_3_, although depending on the ye’elimite to belite and calcium sulphate ratio, also monosulphate, C–S–H gel-like phase, strätlingite and/or hydrogarnet can form [8,14,15,16].

CSA cement is commonly produced by calcination of limestone, Al-rich raw materials (bauxite, Al-clay) at ca. 1250 °C with the addition of the gypsum [3]. Compared with OPC production, the lower clinkering temperature consumes ca. 25% less energy, while the lower need for carbonate reduces the CO_2_ emissions by ca. 25–35% [2]. Additionally, different industrial waste materials have been used for CSA clinker production, like the “red mud” from the Bayer process of alumina production [17,18], phosphogypsum [19], coal combustion fly ash and flue gas desulphurisation wastes [20,21,22,23,24].

In all cases listed above, industrial waste products were treated with the high-temperature calcination step at temperatures >1200 °C, except in the hydrothermal-calcination process where the synthesis temperature is attained at 1050 °C [23]. However, most recently, Paaver et al. [25] have shown that mechanically activated high calcium fly ashes from circulating fluidised bed combustion (CFBC) boilers with high content of free CaO and CaSO_4_, belite and alumosilicate minerals (and Ca–Si–Al-rich glassy phase) behave similar to CSA cement. Activated CFBC ash yielded upon hydration compressive strength up to 30 MPa in 7 days and 60 MPa in 90 days without any need for calcination, chemical activation or blending with other types of binders. The cementitious properties of Ca- and S-rich CFBC ash are controlled, analogous to CSA cement, by the formation of abundant crystalline ettringite and possibly heterogeneous C–(A)–S–H gel-like phase, whereas the ettringite forms in Ca- and sulphate rich CFBC ashes by the reaction of the anhydrite/gypsum and Al-bearing phases (glass, clay minerals and feldspars) [25].

CFBC boilers operate at much lower temperatures (750–900 °C) than the conventional high temperature (>1200 °C) pulverised combustion (PC) boilers and allow efficient firing of low-quality Ca- and S-rich fuels with nearly complete flue gas SO_2_ binding efficiency [26]. The CFBC ashes have been used in mixtures with PC ashes [27] to activate the slag and OPC [28,29,30]. However, the high content of free-CaO and sulphate has limited their wider use [26,31,32]. Hence, the use of the mechanically activated Ca- and sulphate rich CFBC fly ashes as alternative CSA-type cementitious materials would significantly widen their reuse and applicability in different construction and soil mass-stabilisation fields.

The aim of this contribution is to determine the effects of activated fly ash-based calcium sulphoaluminate type (FA-CSA) binder mixtures with OPC on the paste and mortar hydration and strength gain. Hydration and strength development are studied in raw fly ash, FA-CSA and OPC mixtures at different replacement rates and in three-component mixtures of FA-CSA, OPC and sand.

## 2. Materials and Methods

Fresh unhydrated raw total fly ash material was obtained from the ash separation system at Balti Thermal Power Plant CFBC boiler No. 8 in Estonia, which uses oil shale as its primary fuel. Oil shale is a Ca-rich low calorific fuel with high mineral matter content, and ca. 40–50 wt.% of the combusted shale remains as Class C type ash that is mostly (ca. 98% of the ash production) landfilled [33]. The raw oil shale CFBC ash has weak pozzolanic and self-cementitious properties [34,35,36], but the ash has been studied for accelerated carbonation and alkali activation to improve the mechanical properties of the ash aggregates [37,38,39,40,41,42].

The mechanical activation of the fly ash was performed in a dry state with a planetary ball mill (RETCH PM100), using 20 mm-size steel grinding balls in a 500 mL steel grinding jar. Ash was dry-milled for 4 min at a rotation speed of 500 rpm in 0.5 kg batches. A milling time of 4 min was chosen due to loss of milling efficiency and particle agglomeration over longer timespans [25]. Particle size analysis was performed with raw and milled ash using a laser diffraction particle size analyser (Malvern Mastersizer 3000 + Hydro EV). Ethanol was used as a dispersant, and the samples were continually ultrasonicated during measurements.

Pastes of raw ash and mechanically activated Ca-rich fly ash, ordinary Portland cement and mortars with sand (Table 1) were mixed at different ratios (Table 2) with a water/ash ratio of 0.4, were poured into 30 × 30 × 30 mm moulds in three replicas and placed on a vibrating plate for 1 min for compaction. Ordinary Portland cement CM I 42.5R and normalised sand with the grain size of 0.64–2 mm were used to mix with FA-CSA. The mixing was performed in a laboratory environment at the average ambient temperature of 20–22 °C and 50–60% relative humidity. The samples were then left to cure under the same conditions in an open-air environment for 7 and 28 days. The uniaxial compressive strength of pastes and mortars were measured after 7 and 28 days under continuous loading (20 kPa·s^−1^) until the specimen broke.

The chemical composition of the raw materials (Table 1) was determined by means of X-ray fluorescence spectrometry on a Rigaku Primus II XRF spectrometer using the SQX quantification model. The thermogravimetric (DTG)analysis) of selected pastes and pure compounds cured for 28 days were performed on an STA 449 F3 Jupiter thermal analyser in Al_2_O_3_ crucibles by heating to 1000 °C at 10 °C min^−1^.

Isothermal calorimetry was performed using micro reaction calorimeter μRC from Thermal Hazard Technology (THT) to measure the heat released during hydration of FA-CSA and OPC mixture pastes. The pastes were made at a water/solid ratio of 0.4, and measurements were taken at 25 °C for 48 h for mixtures and over the 14 days for the pure FA-CSA paste.

The mineral composition of raw materials and mixed pastes and mortars after 7 and 28 was determined in selected samples by means of X-ray diffraction (XRD) analysis on a Bruker D8 Advance diffractometer in randomly oriented pressed powder samples, using Ni-filtered Cu*K*α radiation and LynxEye linear detector over the 2–70° 2θ region. The mineral composition of samples was interpreted and modelled using the Rietveld algorithm-based code Topaz 4.0. Amorphous phase (glass) content was determined by spiking the samples with 10 wt.% ZnO prior to measurement and calculated from the difference between the added and measured ZnO spike, assuming that the apparent lower ZnO content is caused by the adsorption of X-rays by the amorphous phase. The microstructure and chemical composition of pastes was investigated using Zeiss EVO15MA SEM with Oxford X-MAX energy-dispersive detector.

## 3. Results and Discussion

### 3.1. Composition of the Raw Ash and Hydration Products

**Figure 1:**
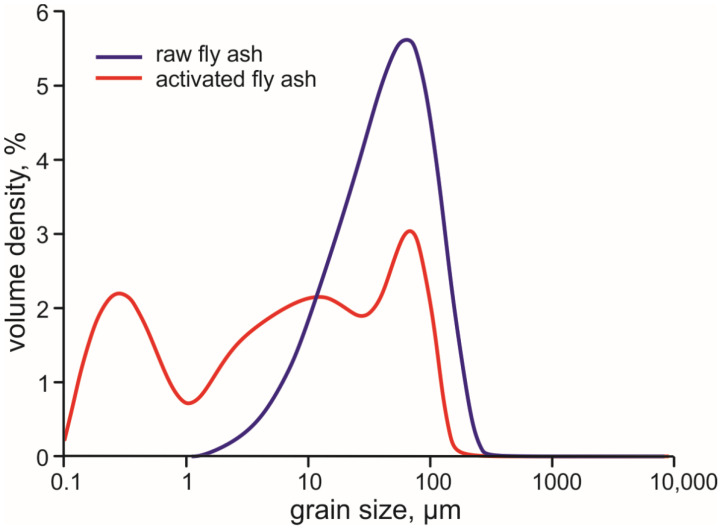
Grain size distribution of the raw and mechanically activated CFBC fly ash.

The mean grain size of the raw fly ash varies between 40–45 µm and is characterised by unimodal, nearly normal grain-size distribution that is slightly skewed towards finer particles with average d_10_, d_50_, d_90_ values of 10, 44 and 110 µm, respectively (Figure 1). The grain size distribution of the mechanically activated ash shows, due to the break-up of the larger particles/aggregates, a significant increase in fine particles, particularly in the <1 µm-size range, and the development of the three-modal grain-size distribution. Paaver et al. [25] have shown that the raw CFBC fly ash comprises irregular-flaky porous lumps and aggregates typically 50–80 µm in size and spherical cenospheres of 1 to 20 µm-size. The mechanical activation causes the large lumps and glassy cenospheres to break up, though the small (<10 µm) cenospheres retain their shape and integrity. The grinding increases the surface area of the particles and enhances, thus, the FA reactivity. In addition, the largely variable grain-sizes of the activated FA enables tighter packing of the particles creating a more compact structure of the paste.

**Figure 2:**
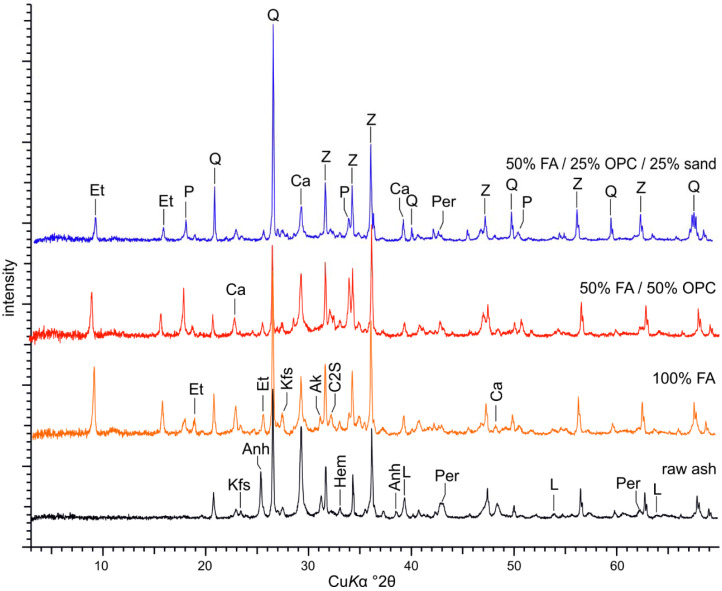
Representative XRD patterns of the activated ash, OPC and sand pastes/mortars after 28 days of curing. Legend for major peaks: Ak—akermanite; Anh—anhydrite; Ca—calcite; C2S—belite; Et—ettringite; Hem—hematite; Kfs—K-feldspar; L—lime; P—portlandite; Per—periclase; Q—quartz; Z—zinc oxide. ZnO spike was added to estimate the amorphous phase content.

The mineral composition of the crystalline phases of the oil shale fly ash (Figure 2) is characterised, first, by the residual primary silicate phases—quartz, K-feldspar, and K-mica. Secondly, by the calcite that has remained in the ash due to incomplete decarbonisation processes in CFBC boilers at temperatures 800–850 °C [43,44] and periclase (MgO) formed by thermal dissociation of dolomite, and lime (CaO_free_) after complete dolomite and partial calcite decomposition. Due to the effective desulphurisation of flue gases, the CFBC fly ash is rich in Ca-sulphate (anhydrite, on average 8–10 wt.%). Additionally, amounts of the secondary Ca- and Ca–Mg silicates (belite—C2S, akermanite and merwinite) forming in boilers by reactions between CaO_free_ and silica [45] are present (Figure 2). The estimated amount of amorphous Ca–Al–Si glass phase in CFBC fly ash varies between 15–20 wt.%. The chemical composition of the activated fly ash is characterised by SiO_2_ and CaO varying between 35–36 wt.% and 28–32 wt.%, respectively (Table 1). The content of Al_2_O_3_, MgO and Fe_2_O_3_ are in the range of 5–6, 8–9 and 2–5 wt.%, respectively. The SO_3_ content varies between 6–9 wt.%.

Due to the rapid slacking of the lime (CaO_free_), the Ca-hydroxide (portlandite) forms in hydrated FA-CSA paste while anhydrite gets completely dissolved (Figure 2). Liira et al. [46] have experimentally shown that the hydration of lime and formation of the portlandite in oil shale CFBC ash occurs together with complete dissolution of anhydrite already during the first 24 h of the hydration. In parallel with anhydrite dissolution, the precipitation of the Ca-sulphoaluminate hydrate phase, ettringite, is initiated and continues for 6–7 days [46]. Paaver et al. [25] showed that ettringite composes up to 20% of minerals in hydrated pastes of the activated FA already after seven days, whereas ettringite abundances up to 40% have been reported in oil-shale processing waste deposits [47,48,49]. Dissolution of anhydrite, slacking of the lime, and formation of abundant ettringite is also evident in FA-CSA mixtures with OPC and sand (Figure 2).

Bernardo et al. [50] have suggested that ettringite formation starts immediately during the first hours of hydration, and that the rate of its precipitation is limited only by the availability of the reactants. Ettringite forms at the expense of the dissolved Ca, sulphate and aluminium [51]. The nascent ettringite precipitated during the first hours of hydration, while the CaO_free_ is still present, occurs in diffuse colloidal form. Still, ettringite quickly develops into typical well-crystalline euhedral hexagonal prismatic fibre like crystallite meshes once the slaking of the lime is completed [52]. Commonly, the Ca-sulphate has been considered a limiting reagent for ettringite formation in CFBC ashes [53]. However, Paaver et al. [25] have noted that ettringite formation in oil-shale FA-CSA is not limited by the sulphate but rather by the availability of the dissolved Al species. The hydrated oil-shale FA is characterised by high pH at ca. 13 that causes natural alkaline activation of the ash and dissolution of Al from Al-bearing aluminosilicate minerals phases (clay minerals, K-feldspar) and amorphous Ca–Al–Si glassy phase present in fly ash [33] that are used up for ettringite formation.

**Figure 3:**
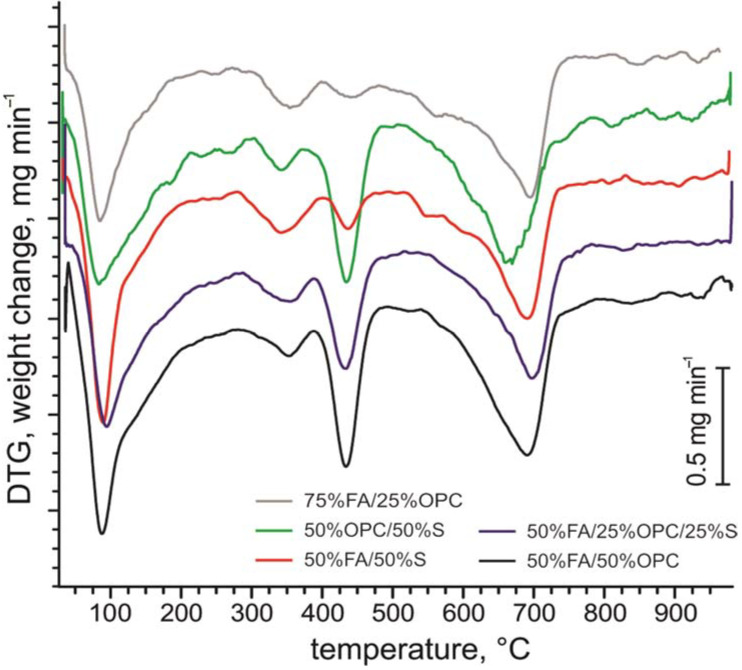
Representative curves of thermogravimetric (DTG) analysis of studied FA-CSA and OPC pastes after 28 days of curing.

The derivative thermogravimetric (DTG) analysis of FA-CSA paste and its mixtures with OPC and sand after 28 days of hydration (Figure 3) shows in DTG curves significant mass loss at temperatures between 100 and 200 °C with a steep endothermic thermal effect. The steepest mass decline at ca. 90–100 °C can be attributed to the free water loss and water loss from ettringite [54].

However, the mass loss with decreasing endothermic effect continuing between temperatures ca. 100 to nearly 250 °C can be associated with decomposition C–S–H gel-like phase and/or some other hydrous semicrystalline amorphous phase, and ettringite [54,55]. Though the overlap of the multitude of semicrystalline phases in pastes makes C–S–H gel-like phase identification solely by thermogravimetric analysis speculative [25], then Leben et al. [33] have shown using nuclear magnetic resonance (NMR) spectroscopy analysis, the presence of abundant C–(A)–S–H type gel-like phase in ash sediments diagenetically altered under naturally alkaline conditions in oil-shale ash waste deposits. A similar process can be suggested for activated fly ash mixtures. The distinct mass loss and endothermic thermal effect at ca. 400–450 °C is due to the dehydroxylation of Ca(OH)_2_ and the mass losses at around 600–680 °C are associated with decomposition of carbonate minerals and escape of CO_2_ [54,56].

**Figure 4:**
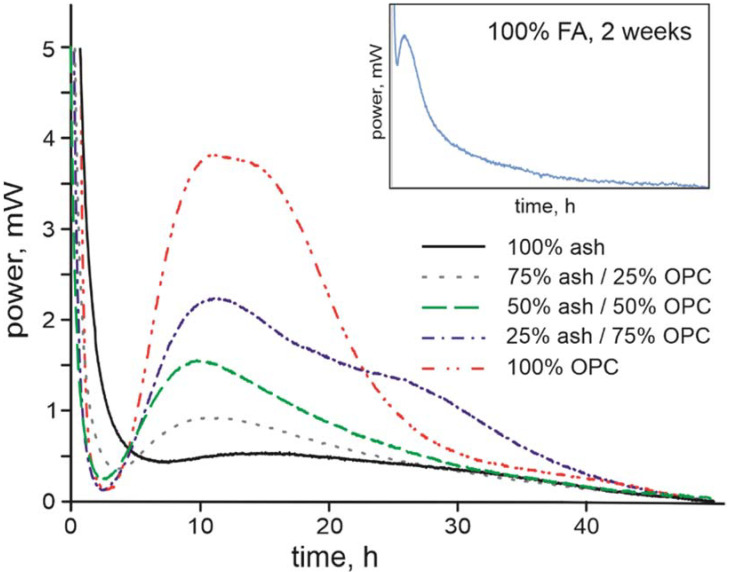
Isothermic calorimetric heat dispersion (mW) graphs of FA-CSA and OPC pastes over 48-h timeframe. The insert shows the heat dispersion from the pure FA-CSA paste measured over two weeks.

The heat curves for the FA-CSA and its mixtures with OPC (Figure 4) show that substitution of OPC with higher amounts of FA-CSA results in progressively lower heat production rate and somewhat reduced kinetics of heat production with the intensity of the heat release peak of the pure FA-CSA paste more than five times lower than in hydration of OPC. A similar phenomenon has been observed for OPC, and raw fly ash and granulated blast slag mixtures [57,58], where the heat production rate was significantly reduced in mixtures with FA and the peak of the heat production appeared slightly later and over a longer period than in OPC hydration. This property of lower heat production can be beneficial in some concrete applications where cracking caused by thermal stresses should be avoided [31].

### 3.2. Compressive Strength of FA-CSA, OPC and Sand Mixtures

**Figure 5:**
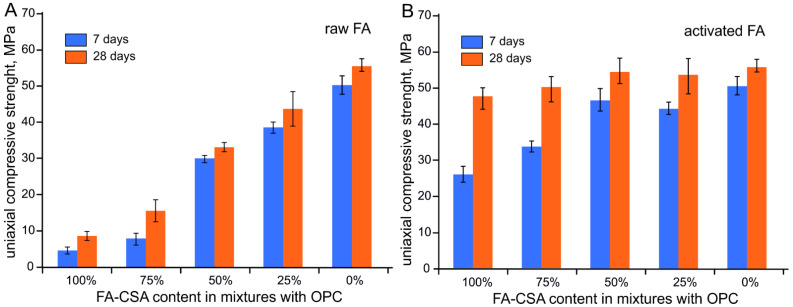
Average uniaxial compressive strength OPC mixture pastes with raw (**A**) and mechanically activated (**B**) fly ash after 7 and 28 days of hydration.

The uniaxial compressive strength of the raw FA paste yields only 4–5 MPa and 8–9 MPa after 7 and 28 days of curing, respectively (Figure 5A), whereas in pastes of raw FA with OPC, the uniaxial compressive strength decreases nearly linearly from on average 58.2 MPa in pure OPC compound to ca. 9 MPa in 100% raw ash after 28 days of curing. The pastes with <50% raw FA substitution to OPC show a slight increase in compressive strength values after seven days of hydration, suggesting activation of the ash-OPC system and some contribution from the ash hydration to the overall strength development at early stages. However, in the long term, the raw FA seems to behave as an inert component.

In OPC pastes with activated FA, in contrast, the 50% OPC substitution by FA-CSA results in nearly the same average uniaxial compressive strength as in pure OPC compound, reaching values 40 MPa and 43 MPa after seven days of hydration, and ca. 54 MPa and 58 MPa after 28 days, respectively (Figure 5B). Interestingly, the average compressive strength of the 50/50 OPC–FA-CSA paste (~40 MPa) is somewhat higher compared with 75/25 OPC–FA-CSA paste (~38 MPa) after seven days of hydration, indicating that FA-CSA addition improves the development of the early strength in mixtures with OPC. Significantly higher compressive strength compared with raw FA pastes were also recorded in pure FA-CSA and the paste with 75% OPC replacement reaching after 28 days of curing ~36 and 42 MPa, respectively (Figure 5B).

**Figure 6:**
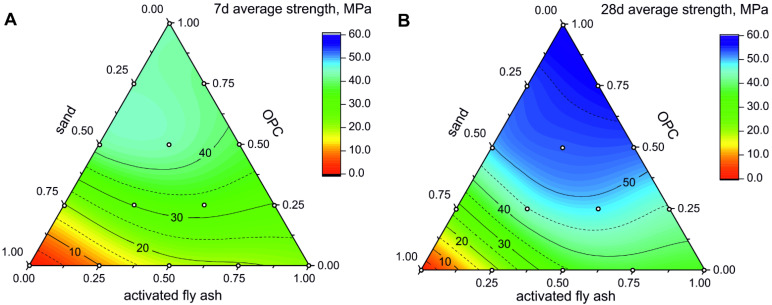
Uniaxial compressive strength of OPC-FA-CSA and sand mixtures after 7 (**A**) and 28 days (**B**) of hydration. Isolines show the uniaxial compressive strength in MPa. Empty circles mark the composition of the measured samples.

For mortars with sand and FA-CSA the mixtures yield after 28 days of curing the average compressive strength of 37.6, 34.7 and 17.6 MPa for 25, 50 and 75% sand content, respectively, whereas the compressive strength reaches 10 MPa or higher values for all mortar mixtures already after seven days of curing (Figure 6A).

It is noteworthy that the compressive strength of the pure FA-CSA and mortars with 25% and 50% of sand yield nearly the same value (36–38 MPa) after 28 days of curing. All mortars with 25%, 50%, and 75% FA-CSA replacement for OPC reach compressive strength values 30 MPa and higher in 7 days and exceed 45 MPa after 28 days (Figure 6). For example, the mortar with a 50/25/25 ratio of sand, OPC and FA-CSA yielded average uniaxial compressive strength of 30.3 MPa after seven days and 44.7 MPa after 28 days of curing. Notably, the reduction in the compressive strength in mixture with 50% FA-CSA replacement of OPC is in the range of 10% compared to the equivalent 50/50 mortar with only OPC and sand, which suggests that FA-CSA can be successfully used for high volume OPC replacement. In comparison, the reported reductions in the compressive strength for mortars with FA replacement to OPC are typically larger than 40% for mixtures with replacement rates higher than 50% [59,60,61].

One of the major drawbacks highlighted for high-volume OPC replacement with FA is its retarded strength development at the early stages of hydration [31,60]. Commonly, during the first weeks of hydration, the fly ash acts like an inert filler that may have only some beneficial properties providing denser packing and seeding effect [31,60]. The pozzolanic reactions between glassy phases in FA and alkaline environment controlled by presence of Ca-hydroxide that produce C–(A)–S–H gels are delayed because of the slow build-up of the alkalinity needed for FA particles dissolution in the pore solution until the pH 12 is reached because the silica hydration is significantly advanced in solutions with pH > 11.8 [62].

**Figure 7:**
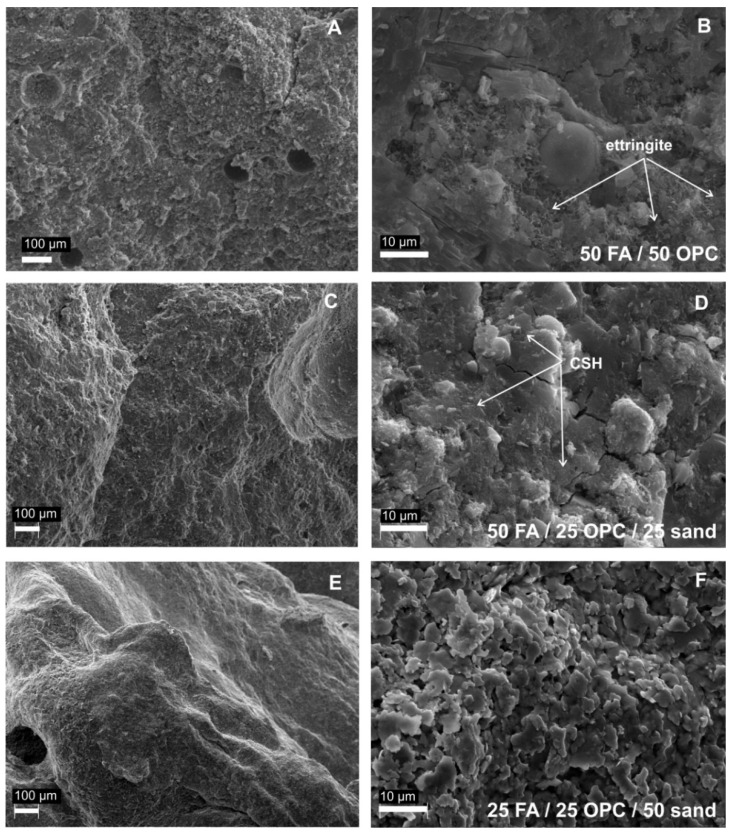
SEM backscattered electron images of pastes after 28 days of curing: (**A**,**B**) 50% CSA-FA/50% OPC mixture; (**C**,**D**) 50% CSA-FA/25% OPC/25% sand mixture; (**E**,**F**) 25% CSA-FA/25% OPC/50% sand mixture.

This propensity of slow hydration in FA-OPC pastes is characteristic to Class F fly ashes rich in silica and aluminium [60]. However, in CFBC ashes, mainly classified as Class C fly ash enriched with Ca and sulphate, the early strength is produced by rapid precipitation of ettringite rather than C–S–H gel formation. CFBC ashes are, thus, devoid of slow reactivity and provide excellent compressive strength already during the first days of the hydration. At later stages, when the rising pH activates the pozzolanic reactions in pore-solution, the formation of the C–(A)–S–H gel-like masses complement to the development of the strength in later stages of the hydration [25], and over time the pastes/mortars achieve strengths comparable to mixtures with OPC. Paaver et al. [25] have shown that the high compressive strength of FA-CSA pastes is associated with the development of a more compact microstructure with mechanically activated fly ash that improves the bonding between the paste particles and the aggregates [63,64,65]. The compact structure of the activated FA pastes and mortars is evident from scanning electron images of the pastes and mortars (Figure 7) showing progressive filling of the pores-space with the dense ettringite meshes and possibly C–(A)–S–H gel-like masses.

## 4. Conclusions

The reduction of the carbon dioxide emissions in cement clinker production and the development of low-carbon binders have become the priorities of the cement industry. Fly-ash-based binders are considered as viable alternatives to CO_2_-extensive cement clinker systems, however, the use of waste ash produced in circulating fluidised bed combustion (CFBC) boilers with high content of free CaO and Ca-sulphate has been hindered mainly by their variable composition and poor cementitious properties. Our study shows that, indeed, the raw Ca- and S-rich oil shale CFBC ash has low self-cementitious properties and acts in mixtures with OPC as an inert filler. However, the mechanical activation of CFBC FA provides a nearly tenfold increase in the compressive strength over raw fly ash and activated fly ash can be used as a novel binder system. The activated CFBC ash behaves similarly to the Ca-sulphoaluminate (CSA) cement, and the rapid formation of ettringite controls the cementitious properties. Our results show that 50% replacement of OPC with FA-CSA results in nearly the same compressive strength (<10% strength reduction) as in pastes with only OPC binder, whereas the development of the early strength is improved. The compact structure of the activated ash pastes and the evolution of dense ettringite crystallite meshes control the strength gain at the early stages of the hydration. The strength development is further complemented with the pozzolanic reactions triggered by the raising pH in pore-solution leading to the formation of the C–(A)–S–H gel-like masses at the later stages. Further studies should aim to test the long-term durability (freeze–thaw cycles, heat resistance, leaching, etc.) of the mechanically activated CFBC fly ash pastes and mortars.

## Figures and Tables

**Table 1:** The Chemical Composition of Raw Materials Used—Fly Ash (FA), Ordinary Portland Cement (OPC) and Sand, wt.%. LOI—Loss on Ignition at 950 °C.

	SiO_2_	Al_2_O_3_	TiO_2_	Fe_2_O_3_	MnO	CaO	MgO	Na_2_O	K_2_O	P_2_O_5_	SO_3_	LOI
FA	35.1	8.5	0.4	3.9	0.1	29.9	5.1	0.1	4.3	0.1	6.8	4.9
OPC	20.6	4.9	0.2	2.1	0.1	63.4	4.0	0.2	1.7	0.6	0.4	1.7
Sand	89.6	3.6	0.05	0.5	0.04	0.4	0.2	0.7	1.3	0.1	0.1	3.2

**Table 2:** Fly ash (FA)—ordinary Portland cement (OPC) and sand mixtures (proportion of compounds in wt.%).

Mixture No.		1	2	3	4	5	6	7	8	9	10	11	12	13	14
content, wt.%	FA	100	75	50	25	0	75	50	25	0	50	25	0	25	0
	OPC	0	25	50	75	100	0	25	50	75	0	25	50	0	25
	sand	0	0	0	0	0	25	25	25	25	50	50	50	75	75

## References

[1] Gartner E.M., Hirao H. (2015). A review of alternative approaches to the reduction of CO_2_ emissions associated with the man-ufacture of the binder phase in concrete. Cem. Concr. Res..

[2] Hanein T., Galvez-Martos J.-L., Bannerman M. (2018). Carbon footprint of calcium sulfoaluminate clinker production. J. Clean. Prod..

[3] Glasser F., Zhang L. (2001). High-performance cement matrices based on calcium sulfoaluminate–belite compositions. Cem. Concr. Res..

[4] Juenger M., Winnefeld F., Provis J., Ideker J. (2011). Advances in alternative cementitious binders. Cem. Concr. Res..

[5] Juenger M.C., Snellings R., Bernal S.A. (2019). Supplementary cementitious materials: New sources, characterization, and performance insights. Cem. Concr. Res..

[6] Chaunsali P., Mondal P. (2015). Influence of Calcium Sulfoaluminate (CSA) Cement Content on Expansion and Hydration Behavior of Various Ordinary Portland Cement-CSA Blends. J. Am. Ceram. Soc..

[7] Hargis C.W., Telesca A., Monteiro P.J.M. (2014). Calcium sulfoaluminate (Ye’elimite) hydration in the presence of gypsum, calcite, and vaterite. Cem. Concr. Res..

[8] Ben Haha M., Winnefeld F., Pisch A. (2019). Advances in understanding ye’elimite-rich cements. Cem. Concr. Res..

[9] Pera J., Ambroise J. (2008). Use of Calcium Sulfoaluminate Cement to Improve Strength of Mortars at Low Temperature.

[10] Péra J., Ambroise J. (2004). New applications of calcium sulfoaluminate cement. Cem. Concr. Res..

[11] Shi W., Shafei B., Liu Z., Phares B.M. (2019). Early-age performance of longitudinal bridge joints made with shrink-age-compensating cement concrete. Eng. Struct..

[12] Pooni J., Robert D., Giustozzi F., Setunge S., Xie Y., Xia J. (2020). Performance evaluation of calcium sulfoaluminate as an alternative stabilizer for treatment of weaker subgrades. Transp. Geotech..

[13] Pooni J., Robert D., Giustozzi F., Setunge S., Xie Y., Xia J. (2020). Novel use of calcium sulfoaluminate (CSA) cement for treating problematic soils. Constr. Build. Mater..

[14] Martin L.H., Winnefeld F., Tschopp E., Müller C.J., Lothenbach B. (2017). Influence of fly ash on the hydration of calcium sulfoaluminate cement. Cem. Concr. Res..

[15] Winnefeld F., Lothenbach B. (2010). Hydration of calcium sulfoaluminate cements - Experimental findings and thermody-namic modelling. Cem. Concr. Res..

[16] Telesca A., Marroccoli M., Pace M.L., Tomasulo M., Valenti G.L., Monteiro P.J.M. (2014). A hydration study of various calcium sulfoaluminate cements. Cem. Concr. Compos..

[17] Žibret G., Teran K., Žibret L., Šter K., Dolenec S. (2021). Building of the Al-Containing Secondary Raw Materials Registry for the Production of Low CO_2_ Mineral Binders in South-Eastern European Region. Sustainability.

[18] Hertel T., Bulck A.V.D., Onisei S., Sivakumar P.P., Pontikes Y. (2021). Boosting the use of bauxite residue (red mud) in cement—Production of an Fe-rich calciumsulfoaluminate-ferrite clinker and characterisation of the hydration. Cem. Concr. Res..

[19] Shen Y., Qian J.S., Huang Y.B., Yang D.Y. (2015). Synthesis of belite sulfoaluminate-ternesite cements with phosphogypsum. Cem. Concr. Compos..

[20] Chen I.A., Juenger M.C. (2012). Incorporation of coal combustion residuals into calcium sulfoaluminate-belite cement clinkers. Cem. Concr. Compos..

[21] Telesca A., Matschei T., Marroccoli M. (2020). Study of Eco-Friendly Belite-Calcium Sulfoaluminate Cements Obtained from Special Wastes. Appl. Sci..

[22] Julphunthong P., Joyklad P. (2019). Utilization of Several Industrial Wastes as Raw Material for Calcium Sulfoaluminate Cement. Materials.

[23] Rungchet A., Chindaprasirt P., Wansom S., Pimraksa K. (2016). Hydrothermal synthesis of calcium sulfoaluminate–belite cement from industrial waste materials. J. Clean. Prod..

[24] Telesca A., Marroccoli M., Tomasulo M., Valenti G.L., Dieter H., Montagnaro F. (2016). Low-CO_2_ Cements from Fluidized Bed Process Wastes and Other Industrial By-Products. Combust. Sci. Technol..

[25] Paaver P., Paiste P., Liira M., Kirsimäe K. (2021). Mechanical Activation of the Ca-Rich Circulating Fluidized Bed Combustion Fly Ash: Development of an Alternative Binder System. Minerals.

[26] Anthony E.J. (1995). Fluidized-Bed Combustion of Alternative Solid Fuels—Status, Successes and Problems of the Technology. Prog. Energy Combust. Sci..

[27] Anthony E.J., Berry E., Blondin J., Bulewicz E., Burwell S. (2003). Advanced ash management technologies for CFBC ash. Waste Manag..

[28] Dung N.T., Chang T.-P., Chen C.-T. (2015). Hydration Process and Compressive Strength of Slag-CFBC Fly Ash Materials without Portland Cement. J. Mater. Civ. Eng..

[29] Lee H.K., Jeon S.M., Lee B.Y., Kim H.K. (2020). Use of circulating fluidized bed combustion bottom ash as a secondary ac-tivator in high-volume slag cement. Constr. Build. Mater..

[30] Siddique S., Kim H., Jang J.G. (2021). Properties of high-volume slag cement mortar incorporating circulating fluidized bed combustion fly ash and bottom ash. Constr. Build. Mater..

[31] Giergiczny Z. (2019). Fly ash and slag. Cem. Concr. Res..

[32] Glinicki M.A., Jóźwiak-Niedźwiedzka D., Dąbrowski M. (2019). The Influence of Fluidized Bed Combustion Fly Ash on the Phase Composition and Microstructure of Cement Paste. Materials.

[33] Leben K., Mõtlep R., Paaver P., Konist A., Pihu T., Paiste P., Heinmaa I., Nurk G., Anthony E.J., Kirsimäe K. (2018). Long-term mineral transformation of Ca-rich oil shale ash waste. Sci. Total Environ..

[34] Pihu T., Arro H., Prikk A., Rootamm R., Konist A., Kirsimäe K., Liira M., Mõtlep R. (2012). Oil shale CFBC ash cementation properties in ash fields. Fuel.

[35] Uibu M., Somelar P., Raado L.M., Irha N., Hain T., Koroljova A., Kuusik R. (2016). Oil shale ash based backfilling concrete—Strength development, mineral transformations and leachability. Constr. Build. Mater..

[36] Raado L.-M., Hain T., Liisma E., Kuusik R. (2014). Composition and Properties of Oil Shale Ash Concrete. Oil Shale.

[37] Paaver P., Paiste P., Kirsimäe K. (2016). Geopolymeric Potential of the Estonian Oil Shale Solid Residues: Petroter Solid Heat Carrier Retorting Ash. Oil Shale.

[38] Paaver P., Paiste P., Mõtlep R., Kirsimäe K. (2017). Self-Cementing Properties and Alkali Activation Of Enefit280 Solid Heat Carrier Retorting Ash. Oil Shale.

[39] Paiste P., Liira M., Heinmaa I., Vahur S., Kirsimäe K. (2016). Alkali activated construction materials: Assessing the alterna-tive use for oil shale processing solid wastes. Constr. Build. Mater..

[40] Paiste P., Külaviir M., Paaver P., Heinmaa I., Vahur S., Kirsimäe K. (2017). Beneficiation of Oil Shale Processing Waste: Secondary Binder Phases in Alkali Activated Composites. Waste Biomass Valorization.

[41] Paaver P., Paiste P., Liira M., Kirsimäe K. (2019). Alkali Activation of Estonian Ca-Rich Oil Shale Ashes: A Synthesis. Oil Shale.

[42] Usta M.C., Yoruk C.R., Hain T., Paaver P., Snellings R., Rozov E., Gregor A., Kuusik R., Trikkel A., Uibu M. (2020). Evaluation of New Applications of Oil Shale Ashes in Building Materials. Minerals.

[43] Bityukova L., Mõtlep R., Kirsimäe K. (2010). Composition of Oil Shale Ashes from Pulverized Firing and Circulating Flu-idized-Bed Boiler in Narva Thermal Power Plants, Estonia. Oil Shale.

[44] Kuusik R., Uibu M., Kirsimäe K., Mõtlep R., Meriste T. (2012). Open-Air Deposition of Estonian Oil Shale Ash: Formation, State of Art, Problems and Prospects for the Abatement of Environmental Impact. Oil Shale.

[45] Taylor H.F.W. (1997). Cement Chemistry.

[46] Liira M., Kirsimae K., Kuusik R., Motlep R. (2009). Transformation of calcareous oil-shale circulating fluidized-bed com-bustion boiler ashes under wet conditions. Fuel.

[47] Mõtlep R., Kirsimäe K., Talviste P., Puura E., Jürgenson J. (2007). Mineral composition of Estonian oil shale semi-coke sediments. Oil Shale.

[48] Sedman A., Talviste P., Kirsimäe K. (2012). The Study of Hydration and Carbonation Reactions and Corresponding Changes in the Physical Properties of Co-Deposited Oil Shale Ash and Semicoke Wastes in a Small-Scale Field Experi-ment. Oil Shale.

[49] Talviste P., Sedman A., Mõtlep R., Kirsimäe K. (2013). Self-cementing properties of oil shale solid heat carrier retorting residue. Waste Manag. Res..

[50] Bernardo G., Telesca A., Valenti G.L., Montagnaro F. (2004). Role of ettringite in the reuse of hydrated fly ash from fluid-ized-bed combustion as a sulfur sorbent: A hydration study. Ind. Eng. Chem. Res..

[51] Min D., Mingshu T. (1994). Formation and expansion of ettringite crystals. Cem. Concr. Res..

[52] Mehta P.K. (1973). Effect of Lime on Hydration of Pastes Containing Gypsum and Calcium Aluminates or Calcium Sul-foaluminate. J. Am. Ceram. Soc..

[53] Anthony E., Bulewicz E.M., Dudek K., Kozak A. (2002). The long term behaviour of CFBC ash–water systems. Waste Manag..

[54] Scrivener K.L., Snellings R., Lothenbach B. (2018). A Practical Guide to Microstructural Analysis of Cementitious Materials.

[55] Zhang Q., Ye G. (2012). Dehydration kinetics of Portland cement paste at high temperature. J. Therm. Anal. Calorim..

[56] Alarcon-Ruiz L., Platret G., Massieu E., Ehrlacher A. (2005). The use of thermal analysis in assessing the effect of temperature on a cement paste. Cem. Concr. Res..

[57] Klemczak B., Batog M., Giergiczny Z., Żmij A. (2018). Complex Effect of Concrete Composition on the Thermo-Mechanical Behaviour of Mass Concrete. Materials.

[58] Batog M., Giergiczny Z. (2017). Influence of mass concrete constituents on its properties. Constr. Build. Mater..

[59] Supit S., Shaikh F., Sarker P. (2014). Effect of ultrafine fly ash on mechanical properties of high volume fly ash mortar. Constr. Build. Mater..

[60] Nwankwo C.O., Bamigboye G.O., Davies I.E.E., Michaels T.A. (2020). High volume Portland cement replacement: A review. Constr. Build. Mater..

[61] Shaikh F.U.A., Supit S.W.M., Sarker P.K. (2014). A study on the effect of nano silica on compressive strength of high volume fly ash mortars and concretes. Mater. Des..

[62] Bellmann F., Stark J. (2009). Activation of blast furnace slag by a new method. Cem. Concr. Res..

[63] Scrivener K.L., John V.M., Gartner E.M. (2018). Eco-efficient cements: Potential economically viable solutions for a low-CO2 cement-based materials industry. Cem. Concr. Res..

[64] Giergiczny Z. (2005). Effect of fly ash from different sources on the properties of hardened cement composites. Silic. Ind..

[65] Marjanović N., Komljenovic M., Baščarević Z., Nikolic V. (2014). Improving reactivity of fly ash and properties of ensuing geopolymers through mechanical activation. Constr. Build. Mater..

